# Expression levels of cleaved caspase-3 and caspase-3 in tumorigenesis and prognosis of oral tongue squamous cell carcinoma

**DOI:** 10.1371/journal.pone.0180620

**Published:** 2017-07-10

**Authors:** Pei-Feng Liu, Yu-Chang Hu, Bor-Hwang Kang, Yu-Kai Tseng, Pi-Chuang Wu, Chi-Chuang Liang, Yu-Yi Hou, Ting-Ying Fu, Huei-Han Liou, I-Chien Hsieh, Luo-Ping Ger, Chih-Wen Shu

**Affiliations:** 1 Department of Medical Education and Research, Kaohsiung Veterans General Hospital, Kaohsiung, Taiwan; 2 Department of Radiation Oncology, Kaohsiung Veterans General Hospital, Kaohsiung, Taiwan; 3 Department of Otorhinolaryngology-Head & Neck Surgery, Kaohsiung Veterans General Hospital, Kaohsiung, Taiwan; 4 Department of Otorhinolaryngology, School of Medicine, National Defense Medical Center, Taipei, Taiwan; 5 Department of Orthopedics, Show Chwan Memorial Hospital, Changhua, Taiwan; 6 Department of Orthopedics, National Cheng Kung University Hospital, Tainan, Taiwan; 7 Department of Nutrition, Kaohsiung Veterans General Hospital, Kaohsiung, Taiwan; 8 Department of Pathology and Laboratory Medicine, Kaohsiung Veterans General Hospital, Kaohsiung, Taiwan; 9 Institute of Biomedical Sciences, National Sun Yat-Sen University, Kaohsiung, Taiwan; University of North Carolina at Chapel Hill School of Medicine, UNITED STATES

## Abstract

Apoptosis plays a dual role in cancer development and malignancy. The role of apoptosis-related caspases in cancer remains controversial, particularly in oral tongue squamous cell carcinoma (OTSCC). In this study, we examined the protein levels of cleaved caspase-3, caspase-3, caspase-8, and caspase-9 on tissue microarrays consisting of samples from 246 OTSCC patients by immunohistochemistry. Wilcoxon signed-rank test indicated that the protein levels of cleaved caspase-3, caspase-3, caspase-8, and caspase-9 in tumor tissues were significantly higher compared to those in adjacent normal tissues (all p<0.001). The expression level of caspase-8 in tumors was elevated in patients with lymph node invasion. Moreover, positive expression of cleaved caspase-3 was associated with shorter disease-free survival (DFS) in OTSCC patients with moderate differentiation and lymph node invasion. Combination of either positive cleaved caspase-3 or higher caspase-3 expression or both was associated with poor DFS. Interestingly, stratification analysis showed that co-expression levels of positive cleaved caspase-3 or/and higher caspase-3 were associated with better disease-specific survival in patients with advanced stages of the disease, such as large tumor size and lymph node invasion, whereas it was associated with poor DFS in OTSCC patients with moderate cell differentiation and small tumor size. Taken together, cleaved caspase-3 and caspase-3/8/9 could be biomarkers for tumorigenesis in OTSCC patients. The co-expression level of cleaved caspase-3 and caspase-3 might be a prognostic biomarker for OTSCC patients, particular in those patients with certain tumor stages and cell differentiation status.

## Introduction

Apoptosis, which is type I programmed cell death, is a process with typical morphological characteristics including cell size reduction, cytoplasm condensation, membrane blebbing, chromatin collapse and DNA fragmentation into oligonucleosomal size pieces [1]. Apoptosis is the main mechanism for the elimination of unnecessary cells during development and homeostasis in normal tissue. Thus, dysfunction of the apoptotic system leads to the pathogenesis of a variety of diseases, including cancer [2, 3]. Apoptosis can be triggered to remove cells with damaged DNA to prevent tumorigenesis in precancerous lesions [4]. An impaired apoptotic mechanism allows cancer cells to survive in suspension and promotes tumor angiogenesis and invasiveness, which are crucial steps for cancer metastasis [5]. Tumor cells also evade apoptotic mechanisms to acquire resistance against treatments, and this results in treatment failure [6, 7]. Several promising targeted therapies inhibit anti-apoptotic proteins and induce apoptosis to treat certain types of cancers [8].

Caspases are a class of cysteine proteases that are mainly divided into two groups according to their functions in apoptosis (caspase-3/6/7/8/9) and inflammation (caspase-1/4/5/12) [9]. The intrinsic and extrinsic pathways of apoptosis involve two major caspase cascades leading to apoptosis [10, 11]. Caspase-8 is induced by members of the tumor necrosis factor (TNF) receptor family, such as TNF receptor 1 and Fas for the extrinsic pathway. In addition, cytochrome c is released from the mitochondria to the cytoplasm in cells in response to intrinsic stimuli and binds to APAF1 to bring about a conformational change that allows these proteins form the apoptosome, which mediates the activation of caspase-9 [12]. Both caspase-8 and caspase-9 are initiator caspases in the apoptotic signaling cascade. Additionally, caspase-3 is a major executioner caspase that is cleaved and activated by both caspase-8 and caspase-9 initiator caspases. Caspase-3 is cleaved at an aspartate residue to yield a p12 and a p17 subunit to form the active caspase-3 enzyme [13]. Active caspase-3 degrades multiple cellular proteins and is responsible for morphological changes and DNA fragmentation in cells during apoptosis [9].

The results from knockout studies show that mice lacking caspase-3/8/9 do not survive, whereas mice lacking the other caspases develop normally [9], suggesting that the roles of caspase-3/8/9 in physiological development are crucial. Regarding the roles of caspase-3/8/9 in cancer, impaired expression of caspase-8 and caspase-3 can promote tumor formation and progression and treatment resistance in several types of cancer, including oral cancer [14, 15]. However, the expression levels of caspase-8 and caspase-9 are higher in oral squamous carcinoma cells (OSCCs) than in the adjacent normal cells [16]. Moreover, high levels of cleaved caspase-3 contribute to poor disease-free survival in patients with head and neck cancer and breast cancer in response to radiotherapy [17]. In addition, cleaved caspase-3 promotes chemical- or radiation-induced DNA damage and oncogenesis [18]. These results indicate that the roles of caspase-3/8/9 in the development and prognosis of cancer are still controversial.

To date, no study has been carried out using tissue microarrays consisting of a series of surgically resected OTSCC tissues and corresponding tumor adjacent normal tissues to evaluate the difference in the protein levels of cleaved caspase-3 and caspase-3/8/9. In our present study, we investigated whether the expression status of cleaved caspase-3 and caspase-3/8/9 is significantly different between tumor tissues and tumor adjacent normal tissues in OTSCC patients. We also evaluated the relationship of the protein levels with demographic characteristics and pathological outcomes. The effects of cleaved caspase-3 and caspase-3 on the survival of OTSCC patients and stratified groups according to cell differentiation and pathological stages were further investigated to determine whether their suitability as biomarkers for OTSCC patients.

## Materials and methods

### Patients and tissue subjects

The study population comprised 246 samples from patients with pathologically confirmed tongue cancer who underwent biopsy or surgical resection at the Kaohsiung Veterans General Hospital (KSVGH) between 1991 and 2010. The survival time was estimated from the time of surgery to 2013. Pathological TNM classification was determined at the time of initial resection of the tumor in accordance with the guidelines of the 2002 American Joint Committee on Cancer (AJCC). Our data showed that OTSCC patients with moderate (p = 0.023) or poor differentiation (p<0.001); pathological stage of II (p = 0.019), III (p<0.001), or IV (p<0.001); T stage of T2 (p = 0.006), T3 (p<0.001) or T4 (p<0.001); N stage of N1 (p<0.001) or N2 (p<0.001); post-operative radiotherapy (p = 0.001); and post-operative chemotherapy (p = 0.002) had significantly worse disease-specific survival (DSS) (S1 Table). All participants have been verbal explained about the study and signed informed consent. The study was approved by the Institutional Review Board at the KSVGH (IRB number: VGHKS96-CT1-08).

### Specimen characteristics and tissue microarray construction

The specimens were fixed in 10% buffered formalin for one to several days and were then embedded in paraffin wax. Tissue microarray (TMA) blocks were constructed in this study. Each TMA consisted of 129 cores that were 1.5 mm in diameter, including 43 trios, with each trio containing two cores from the tumor tissue and one core from the non-cancerous epithelium of the same patient. The morphology of non-cancer epithelium, identified by senior oral pathologist Dr. Ting-Ying Fu, was the same as that of normal oral epithelium, which excluded pre-cancerous cells with pathological morphology of dysplasia, hyperplasia and hyperkeratosis. The non-cancerous epithelium is termed tumor adjacent normal tissues in this study.

### Immunohistochemistry (IHC)

Rabbit monoclonal anti-caspase-3 antibody for the cleaved form (9661, Cell Signaling Technology, Beverly, MA), rabbit monoclonal anti-caspase-3 antibody for the pro and cleaved forms (sc-7148, Santa Cruz Biotechnology, Inc., Santa Cruz, CA), rabbit polyclonal anti-caspase-8 antibody for the pro and cleaved forms (IMG-5703, IMGENEX or NB100-56116, Novus Biologicals), and rabbit polyclonal anti-caspase-9 antibody for the pro and cleaved forms (sc-7885, Santa Cruz Biotechnology) were utilized for immunohistochemistry using a polymer-based system (Novocastra Novo-Link Max Polymer Detection System, Leica Biosystems) according to the manufacturer’s instructions.

TMA blocks were cut into 4-μm-thick serial sections, deparaffinized in xylene, rehydrated in an ethanol gradient and dipped in PBS. Antigen retrieval was performed by immersion in Tris-EDTA (10 mM, pH 9.0) for 10 min at 125°C in a pressure cooker. Endogenous peroxidase activity was blocked with 3% hydrogen peroxide in methanol for 30 min. The slides were then incubated overnight at 4°C in a humid chamber with anti-cleaved-caspase-3 antibody (dilution 1:100), anti-caspase-3 antibody for the pro and cleaved forms (dilution 1:100), anti-caspase-8 (dilution 1:100) or anti-caspase-9 (dilution 1:30) antibodies. After the slides were washed in phosphate-buffered saline, the slides were developed with a solution of 0.03% diaminobenzidine and counterstained with hematoxylin.

### IHC analysis and scoring

**Fig 1:**
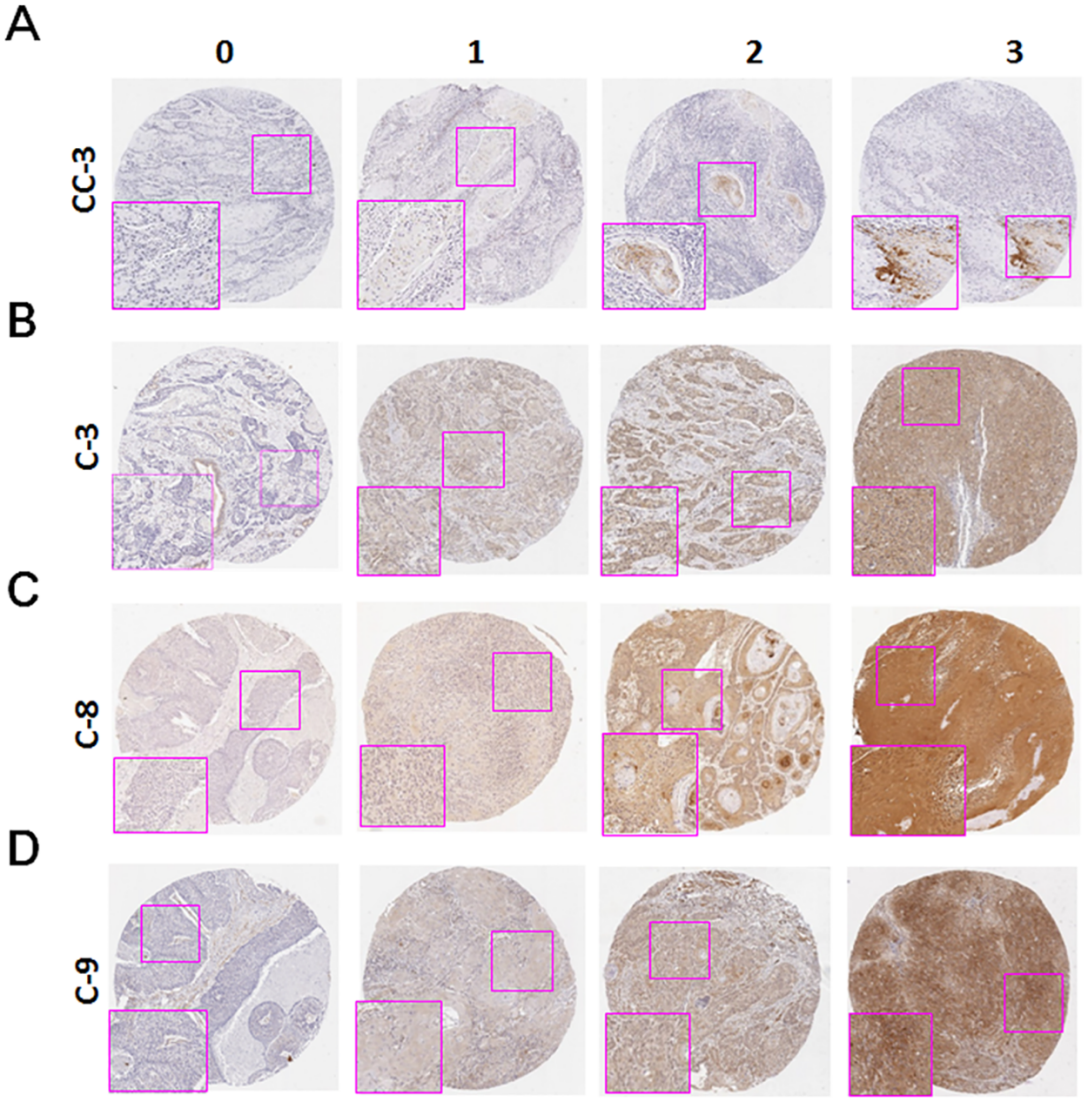
Protein levels of caspases in OTSCC. The staining intensity of cleaved caspase-3 (CC-3, A), caspase-3 (C-3, B), caspase-8 (C-8, C) and caspase-9 (C-9, D) in the OTSCC tissues were scored according to the representative slides. 0 = no signal; 1 = mild; 2 = moderate; and 3 = strong.

Initially, an oral cancer pathologist (Dr. Ting-Ying Fu) and two senior pathology technicians blinded to the clinical data evaluated the slides until all the discrepancies were resolved. Furthermore, both pathology technicians independently reviewed the slides, except the cores with incorrect or uncertain contents, which were scored by the pathologist. The estimated average staining intensity was scored as follows: 0, no signal; 1, mild; 2, moderate; and 3, strong (Fig 1). The percentage of stained cells at each intensity level was graded as 0 (<5%), 1 (5–25%), 2 (26–50%), 3 (51–75%), and 4 (>75%). The intensity score and percentage of positive cells were then added to produce the final scores (0–7). In the final evaluation, the agreement of the caspase scores from the pathologist and technicians was at least 96% (cleaved caspase-3: 99%; caspase-3: 97%; caspase-8: 97%; caspase-9: 96%) in this study. For survival analysis, the expression of caspases was dichotomized to low expression and high expression, with a cutoff set at the 25th percentile to ensure that each group had enough cases. However, the cutoff percentage for each caspase was varied; depending on the distribution of the protein score (S2 Table). The cutoff values were 0, 4, 6, and 5 for cleaved caspase-3, caspase-3, caspase-8, and caspase-9, respectively, in the OTSCC tissues. The cutoff value of 0 represented negative expression for cleaved caspase-3, while cutoff value of >0 was defined as positive expression. For the other caspases, the values ≤ the cutoff point were considered low expression and the others were considered high expression. The results may have been biased for those cases that were close to the cutoff. Nevertheless, we had to arbitrarily include those cases in the high or low expression groups to have fair results.

### Statistical analysis

Student’s *t*-test, Mann-Whitney *U*-test, ANOVA and Kruskal-Wallis one-way ANOVA were used to evaluate the correlation between the expression of each protein and demographic or pathological parameters. Wilcoxon signed-rank test was used to evaluate the different levels of caspases between the tumors and corresponding tumor adjacent normal tissues. DSS was measured from the time of initial resection of the primary tumor to the date of cancer-related death or last follow-up. Disease-free survival (DFS) was calculated from the date of initial resection of the primary tumor to the date of local or regional recurrence or last follow-up. Cumulative survival curves were estimated using the Kaplan-Meier method. Comparison of the survival curves was performed by both the log-rank test (for survival curves) and univariate Cox proportional hazards model (for crude hazard ratio). A multivariate Cox proportional hazards model was used to determine the independent predictors of survival using significant factors from univariate analysis as covariates. A value of p<0.05 (2-sided) was considered significant. The raw clinical data along with caspase expression scores are provided in the supporting information (S3 Table).

## Results

### The association of cleaved caspase-3, caspase-3, caspase-8 and caspase-9 with tumorigenesis and clinicopathological outcomes of OTSCC patients

To compare the expression levels of cleaved caspase-3 and caspase-3/8/9 in OTSCC patients, the tumor tissues and tumor adjacent normal tissues were stained by immunohistochemistry for the determination of protein levels. The representative staining intensity of the expression of each caspase is shown in Fig 1. Cleaved caspase-3, caspase-3, caspase-8, and caspase-9 were only expressed in the cytoplasm of cells. The expression levels of cleaved caspase-3, caspase-3, caspase-8, and caspase-9 in tumor and adjacent normal cells in patients were further scored (Table 1). In contrast to that in the tumor adjacent normal tissue, the expression levels of cleaved caspase-3, caspase-3, caspase-8 and caspase-9 were significantly higher in tumor tissues (all p<0.001, Wilcoxon signed-rank test), suggesting that these caspases might be associated with tumor transformation. Regarding the impact of these caspases on demographical characteristics and pathological outcomes, expression level of cleaved caspase-3 was lower in tumor tissues with poor cell differentiation compared to tumor tissues with moderate cell differentiation (p = 0.024, Table 2). A low level of caspase-3 was correlated with younger (≤40 years old) patients (p = 0.023), whereas a low level of caspase-8 was correlated with older (>50 years old) patients (p = 0.003) and N0 classification (p = 0.017). A high level of caspase-9 was correlated with female patients (p = 0.019), while expression level of caspase-9 was higher in tumor tissues with poor cell differentiation compared to those with well or moderate cell differentiation (p = 0.007).

**Table 1:** The comparisons of cleaved caspase-3 (CC-3), caspase-3 (C-3), caspase-8 (C-8) and caspase-9 (C-9) expression in the tumor and tumor adjacent normal tissues of OTSCC patients.

Variables	No.	Tumor adjacent normal		Tumor		Z	*p*-value*
		mean±SD	Median	mean±SD	Median		
CC-3	203	0.03±0.27	0.00	1.18±1.17	1.00	9.514	**<0.001**
C-3	204	3.49±1.54	4.00	4.70±1.45	5.00	8.037	**<0.001**
C-8	200	6.17±1.25	7.00	6.62±0.64	7.00	5.806	**<0.001**
C-9	211	4.65±1.89	5.00	5.44±1.17	6.00	5.856	**<0.001**

Abbreviations: TSCC, tongue squamous cell carcinoma; SD, standard deviation.

*p-values were estimated by Wilcoxon signed-rank test

**Table 2:** Association of cleaved caspase-3 (CC-3), caspase-3 (C-3), caspase-8 (C-8) and caspase-9 (C-9) expression levels with demographical characteristics and pathological outcomes of OTSCC patients (base of tongue was excluded).

Variable	No. (%)	CC-3			C-3			C-8			C-9		
		Mean±SD	Median	*p value*	Mean±SD	Median	*p value*	Mean±SD	Median	*p value*	Mean±SD	Median	*p value*
Sex													
Female	29 (11.8)	0.86±1.09	0.00	0.115*	4.93±1.33	5.00	0.369*	6.76±0.51	7.00	0.246^†^	5.93±0.84	6.00	**0.019**^†^
Male	217 (88.2)	1.23±1.17	1.00		4.67±1.47	5.00		6.60±0.65	7.00		5.37±1.19	5.00	
Age, y													
≦40	47 (19.1)	1.19±1.14	1.00	0.965^‡^	4.28±1.42^a^^b^	4.00	**0.023**^§^	6.68±0.59^c^	7.00	**0.003**^§^	5.62±1.13	6.00	0.578^‡^
41–50	79 (32.1)	1.19±1.18	1.00		4.57±1.64	5.00		6.80±0.46^d^^e^	7.00		5.47±1.16	6.00	
51–60	67 (27.2)	1.22±1.08	1.00		5.01±1.29^a^	5.00		6.57±0.68^d^	7.00		5.31±1.17	5.00	
>60	53 (21.5)	1.11±1.30	1.00		4.89±1.28^b^	5.00		6.38±0.77^c^^e^	7.00		5.40±1.21	5.00	
Cell differentiation													
Well	26 (10.6)	1.08±1.09	1.00	**0.024**^§^	5.04±1.22	5.00	0.281^‡^	6.54±0.71	7.00	0.642^§^	5.31±1.19^g^	5.50	**0.007**^‡^
Moderate	203 (82.5)	1.26±1.19^f^	1.00		4.64±1.47	5.00		6.62±0.64	7.00		5.38±1.17^h^	5.00	
Poor	17 (6.9)	0.47±0.72^f^	0.00		5.00±1.46	5.00		6.76±0.44	7.00		6.29±0.77^g^^h^	6.00	
AJCC pathological stage													
I, II	167 (67.9)	1.17±1.16	1.00	0.856*	4.65±1.41	5.00	0.428*	6.57±0.66	7.00	0.071^†^	5.43±1.19	5.00	0.787*
III, IV	79 (32.1)	1.20±1.18	1.00		4.81±1.54	5.00		6.72±0.58	7.00		5.47±1.13	6.00	
T classification													
T1, T2	194 (78.9)	1.21±1.17	1.00	0.461*	4.70±1.41	5.00	0.878*	6.61±0.64	7.00	0.517*	5.45±1.19	5.50	0.706*
T3, T4	52 (21.1)	1.08±1.17	1.00		4.73±1.62	5.00		6.67±0.62	7.00		5.38±1.11	6.00	
N classification													
N0	195 (79.3)	1.14±1.13	1.00	0.302*	4.70±1.43	5.00	0.902*	6.57±0.66	7.00	**0.017**^†^	5.38±1.19	5.00	0.154*
N1, N2	51 (20.7)	1.33±1.31	1.00		4.73±1.55	5.00		6.80±0.49	7.00		5.65±1.07	6.00	

Abbreviations: AJCC, American Joint Committee on Cancer.

*p values were estimated by student’s T test.

^†^p values were estimated by Mann-Whitney U test.

^‡^ p values were estimated by 1-way ANOVA test.

^**§**^p values were estimated by Kruskal-Wallis 1-way ANOVA test.

^a^p = 0.003;

^b^p = 0.021;

^c^p = 0.032;

^d^p = 0.026;

^e^p<0.001;

^f^p = 0.007;

^g^p = 0.024;

^h^p = 0.008.

### The relationship of cleaved caspase-3, caspase-3, caspase-8 and caspase-9 with the survival of OTSCC patients

**Fig 2:**
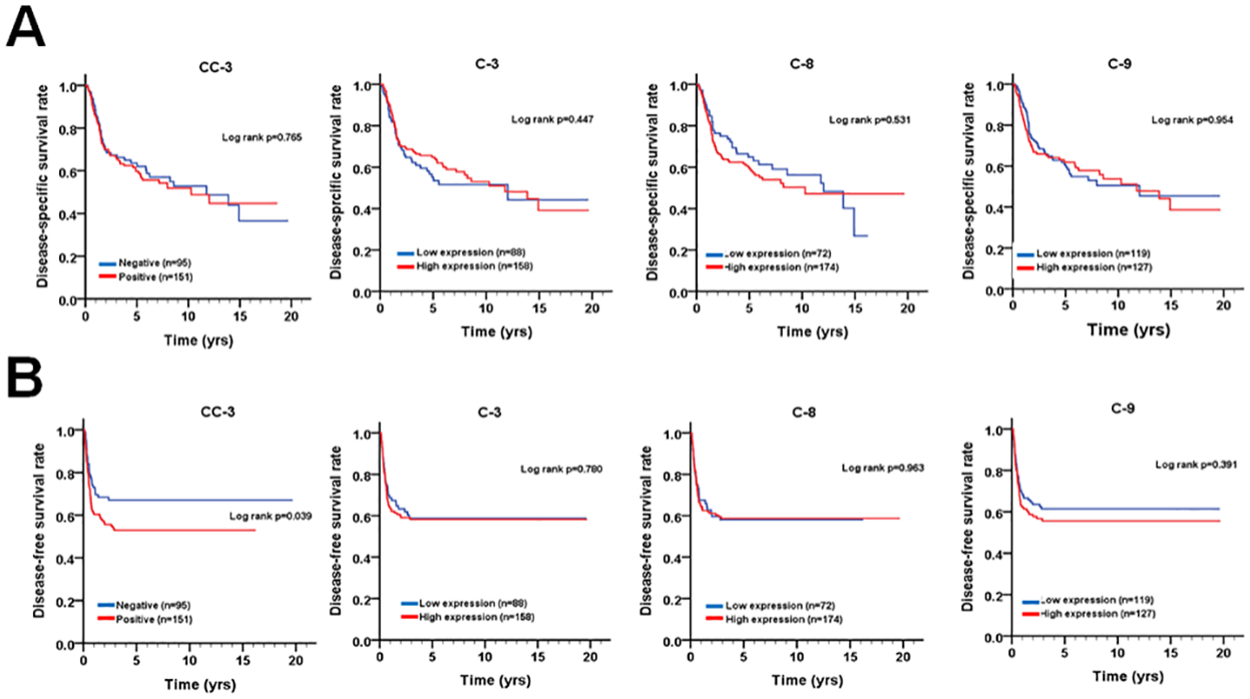
The survival curves of OTSCC patients according to caspase expression. (A) DSS and (B) DFS curves according to the expression levels of cleaved caspase-3 (CC-3), caspase-3 (C-3), caspase-8 (C-8), and caspase-9 (C-9) are shown. The cutoff criteria to split the high expression and low expression of each protein are described in the materials and methods.

**Fig 3:**
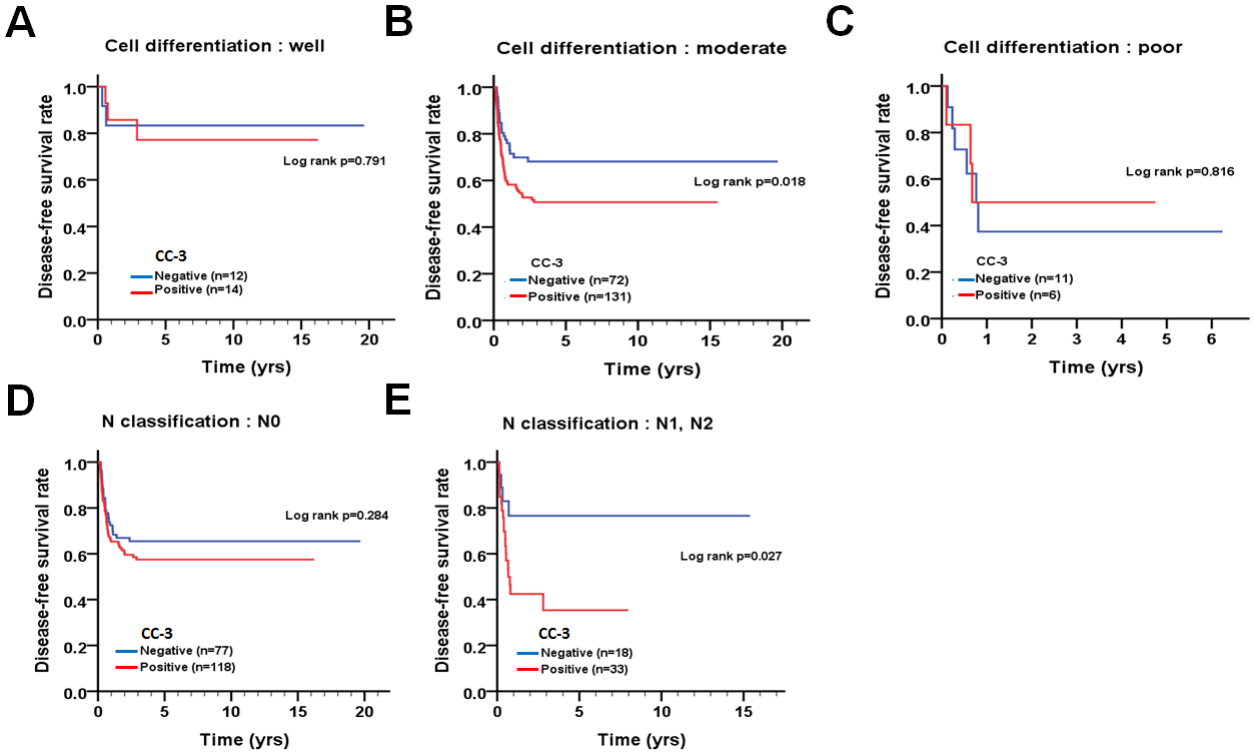
DFS curves of patients with OTSCC according to the pattern of cleaved caspase-3 expression for patients stratified according to the cell differentiation status. Level of expression of cleaved caspase-3 in patients with (A, B and C) different cell differentiation or (D and E) lymph node invasion was evaluated for determination of DFS.

To inspect the relationship of cleaved caspase-3 and caspase-3/8/9 with survival in patients, the protein levels of these caspases were evaluated using univariate and multivariate Cox proportional hazards models (Table 3). The tumor tissues with positive cleaved caspase-3 staining were associated with a shorter DFS in univariate analysis (Table 3, crude hazard ratio [CHR] = 1.57, 95% CI 1.02–2.41, p = 0.041), but this association was not statistically significant in the multivariate analysis (Table 3, adjusted hazard ratio [AHR] = 1.54, 95% CI 1.00–2.40, p = 0.053). Additionally, the levels of caspase-3/8/9 in tumor tissues were not associated with DSS or DFS of patients by univariate and multivariate analyses. Indeed, Kaplan-Meier analysis showed that positive expression of cleaved caspase-3 was associated with shorter DFS but not with DSS (Fig 2). Since positive expression of cleaved caspase-3 was associated with shorter DFS in patients, we further stratified the patients into different groups based on their tumor cell differentiation (well, moderate and poor) and N stage (N0 and N1+N2) for DFS (Fig 3). When the patients were stratified by cell differentiation, positive expression of cleaved caspase-3 was associated with shorter DFS in patients with moderately differentiated tumors (Fig 3A, 3B and 3C, log-rank test p = 0.018). In contrast, for patients with well or poor differentiated tumors, no significant difference related to differential cleaved caspase-3 expression was found. In the stratified analysis for different status of lymph node invasion, patients with lymph node invasion had a significantly shorter DFS when they expressed cleaved caspase-3 compared to those with negative expression of cleaved caspase-3 (Fig 3D and 3E, log-rank test p = 0.027). However, in patients without lymph node invasion, no significant difference related to differential cleaved caspase-3 expression was found (p = 0.284). These results suggest that cleaved caspase-3 might be involved in tumor relapse, particularly in patients with moderately differentiated and lymph node-invaded tumors.

**Table 3:** The expression levels of cleaved caspase-3 (CC-3), caspase-3 (C-3), caspase-8 (C-8) and caspase-9 (C-9) for disease-specific survival and disease-free survival of OTSCC patients.

Variable		Disease-specific survival					Disease-free survival			
		No. (%)	CHR (95% CI)	*p value*	AHR (95% CI)	p value*	CHR (95% CI)	*p value*	AHR (95% CI)	*p value*^†^
CC-3 expression	Negative	95 (38.6)	1.00		1.00		1.00		1.00	
	Positive	151 (61.4)	1.06 (0.72–1.56)	0.765	1.04 (0.70–1.54)	0.848	**1.57 (1.02–2.41)**	**0.041**	1.54 (1.00–2.40)	0.053
C-3 expression	Low	88 (35.8)	1.00		1.00		1.00		1.00	
	High	158 (64.2)	0.86 (0.58–1.27)	0.448	0.74 (0.49–1.10)	0.134	1.06 (0.70–1.61)	0.781	1.07 (0.70–1.63)	0.760
C-8 expression	Low	72 (29.3)	1.00		1.00		1.00		1.00	
	High	174 (70.7)	1.14 (0.76–1.72)	0.532	0.98 (0.65–1.49)	0.930	1.01 (0.65–1.56)	0.963	0.94 (0.61–1.46)	0.787
C-9 expression	Low	119 (48.4)	1.00		1.00		1.00		1.00	
	High	127 (51.6)	1.01 (0.69–1.47)	0.954	0.90 (0.61–1.33)	0.593	1.19 (0.80–1.78)	0.391	1.19 (0.79–1.79)	0.418

Abbreviations: CHR, crude hazard ratio; CI, confidence interval; AHR, adjusted hazard ratio.

*p-value were adjusted for cell differentiation(moderate, poor vs. well), AJCC pathological stage (stage II, III, IV vs stage I), postoperative RT by multiple Cox‘ s regression.

^†^p-value were adjusted for cell differentiation(moderate, poor vs. well), AJCC pathological stage (stage II, III, IV vs stage I) by multiple Cox‘ s regression.

### The association of the combined expression of cleaved caspase-3 and caspase-3 with the survival of OTSCC patients in the stratification model

**Fig 4:**
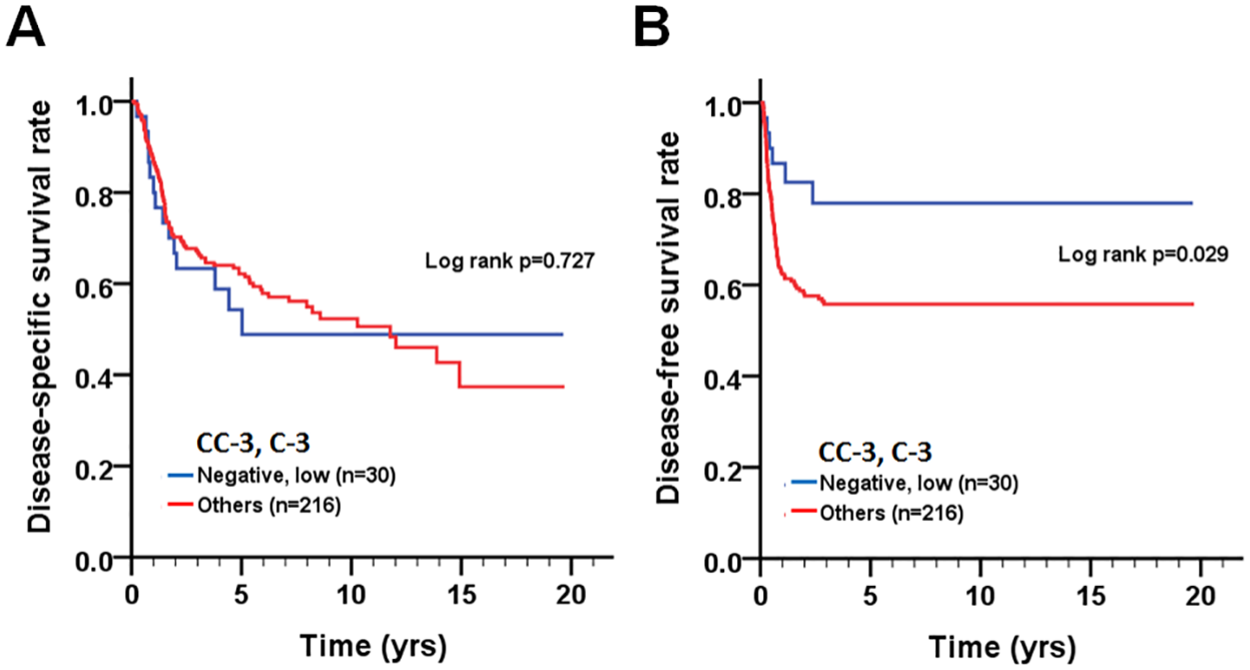
Survival curves of patients with OTSCC according to the combined expression pattern of cleaved caspase-3 and caspase-3. Kaplan-Meier plots were used to analyze effect of the level of co-expression of cleaved caspase-3 and caspase-3 on DSS (A) and DFS (B).

Since caspase-3 can be cleaved to give rise to the active form, we further examined if the combined expression pattern of cleaved caspase-3 and caspase-3 is associated with the prognosis of OTSCC patients. Cox regression analysis showed that patients with a combination of co-expression levels of positive cleaved caspase-3 or/and higher caspase-3 (cleaved caspase-3-negative/caspase-3-high, cleaved caspase-3-positive/caspase-3-low, and cleaved caspase-3-positive/caspase-3-high) had significantly shorter DFS ([CHR] = 2.43, 95% CI 1.06–5.56, p = 0.035) compared to patients with cleaved caspase-3-negative/caspase-3-low expression (Table 4), even after adjusting for cell differentiation (moderate/poor vs. well) and AJCC pathological stage (stage II, III, or IV vs. stage I) ([AHR] = 2.33, 95% CI 1.01–5.35, p = 0.047). Likewise, Kaplan–Meier plots were used to analyze the association of co-expression of cleaved caspase-3 and caspase-3 with DSS and DFS (Fig 4). Co-expression levels of either positive cleaved caspase-3 or higher caspase-3 or both were associated with poor DFS (p = 0.029).

**Table 4:** The co-expression levels of cleaved caspase-3 (CC-3) and caspase-3(C-3) for disease-specific survival and disease-free survival of OTSCC patients.

Variable	No. (%)	Disease-specific survival				Disease-free survival			
		CHR (95% CI)	*p value*	AHR (95% CI)	p value*	CHR (95% CI)	*p value*	AHR (95% CI)	*p value*^†^
CC-3, C-3 expression									
Negative, Low	30 (12.2)	1.00		1.00		1.00		1.00	
Negative, High	65 (26.4)	0.91 (0.52–1.59)	0.727	0.62 (0.35–1.10)	0.104	**2.43 (1.06–5.56)**	**0.035**	**2.33 (1.01–5.35)**	**0.047**
Positve, Low	58 (23.6)								
Positve, High	93 (37.8)								

Abbreviations: CHR, crude hazard ratio; CI, confidence interval; AHR, adjusted hazard ratio.

*p-value were adjusted for cell differentiation(moderate, poor vs. well), AJCC pathological stage (stage II, III, IV vs stage I), postoperative RT by multiple Cox‘ s regression.

^†^p-value were adjusted for cell differentiation(moderate, poor vs. well), AJCC pathological stage (stage II, III, IV vs stage I) by multiple Cox‘ s regression.

**Fig 5:**
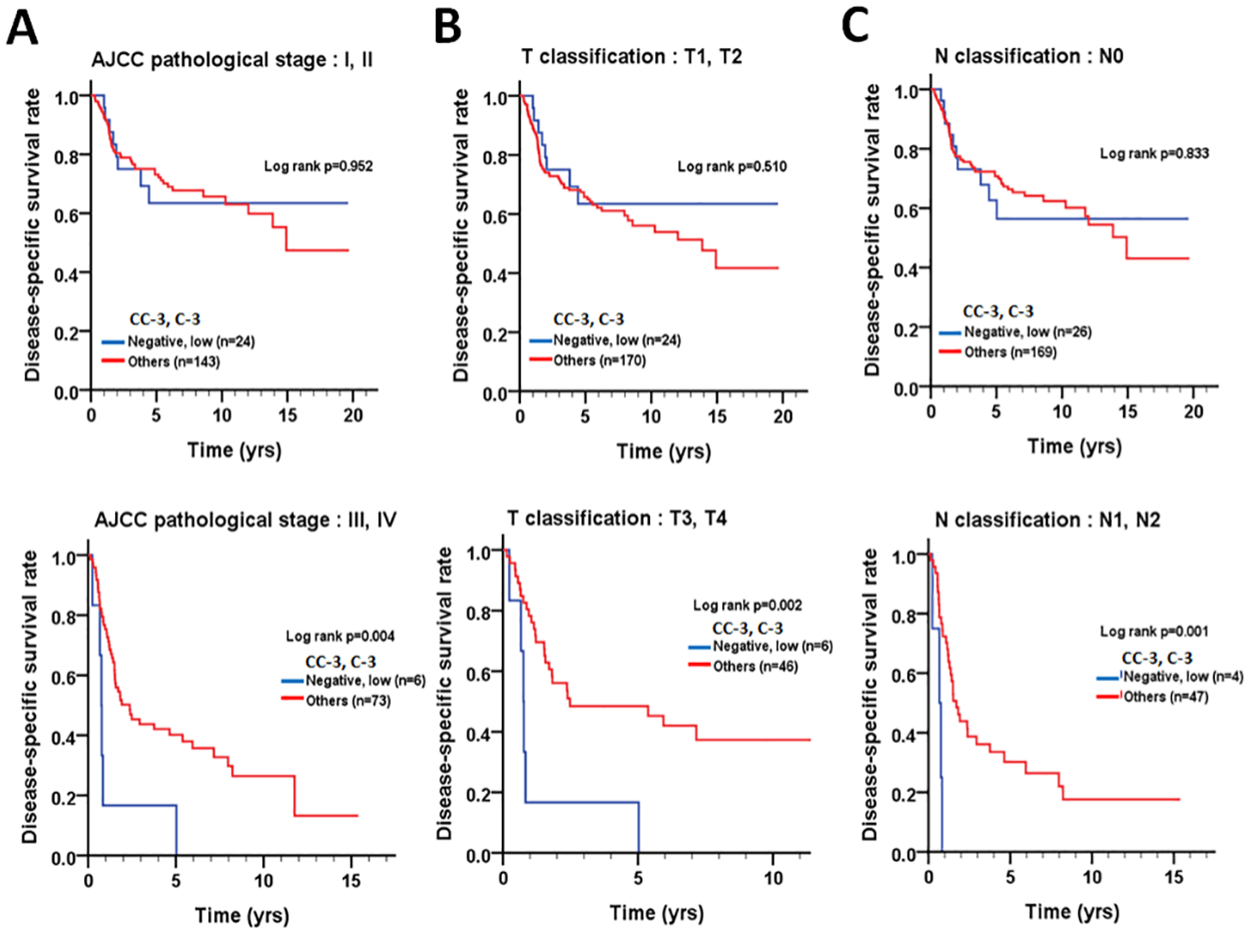
Stratified DSS curves of OTSCC patients according to the level of co-expression of cleaved caspase-3 and caspase-3. Patterns of protein expression in patients stratified according to the AJCC pathological stage (A), T classification (B) and lymph node invasion (C) were used to analyze DSS.

**Fig 6:**
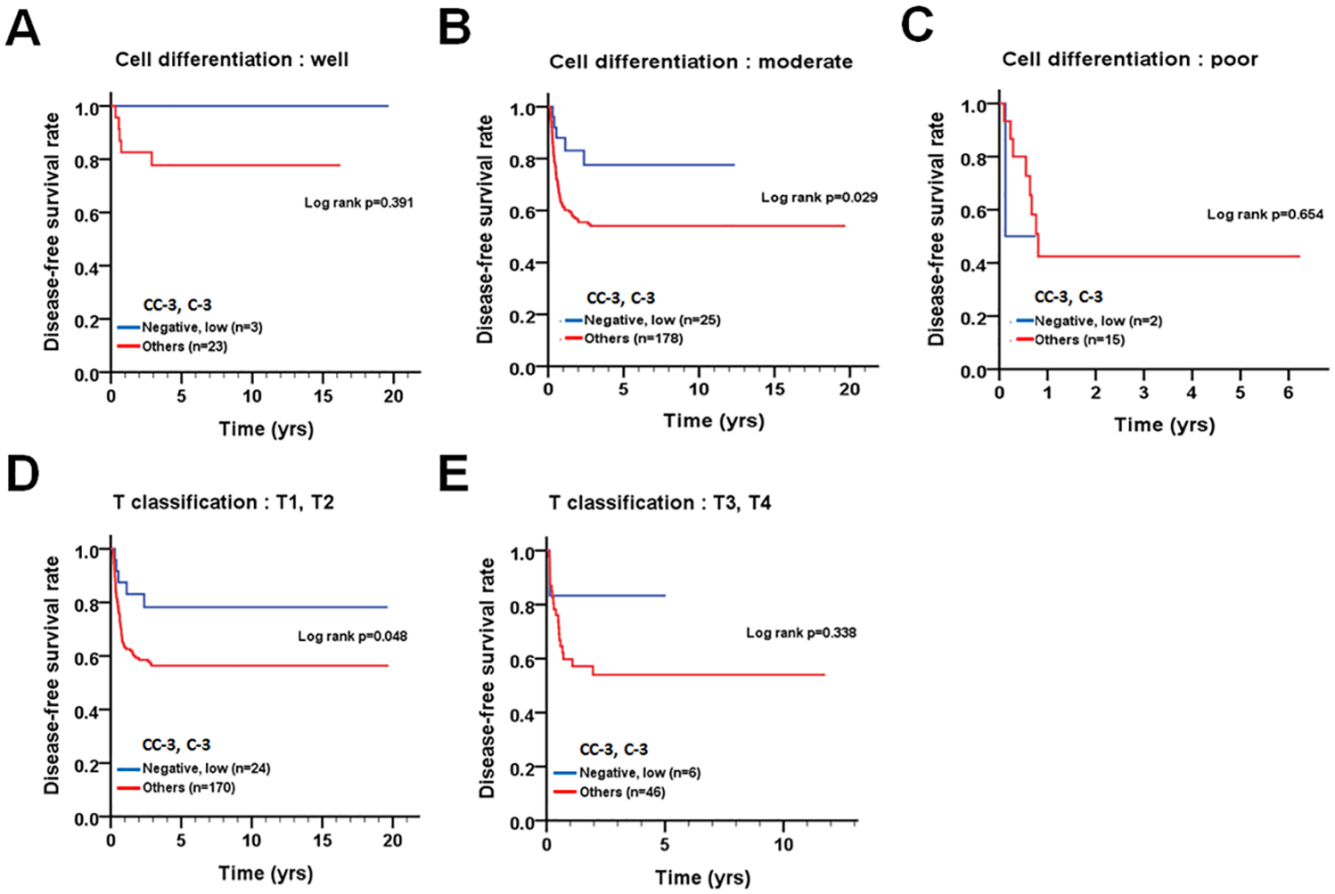
Stratified DFS curves of OTSCC patients according to the level of co-expression of cleaved caspase-3 and caspase-3. Patterns of protein expression in patients with (A) well differentiated tumors, (B) moderately and (C) poorly differentiated tumors, (D) T1+T2 classification and (E) T3+T4 classification.

We further stratified the patients into two groups based on pathological factors, such as cell differentiation (well and moderate/poor), AJCC pathological stage (I = II and III+IV), T stage (T1+T2 and T3+T4), and N stage (N0 and N1+N2) to evaluate the relationship between the levels of the two proteins and DSS or DFS. In patients with advanced stage (AJCC pathological stage III + IV, p = 0.004), large tumor size (T3 + T4, p = 0.002), or lymph node invasion (N1+N2, p = 0.001), co-expression levels of positive cleaved caspase-3 or/and higher caspase-3 or both were significantly associated with longer DSS compared to those with negative cleaved caspase-3 and lower caspase-3 expression (Fig 5). In contrast, patients with moderate cell differentiation (p = 0.029) or small tumor size (T1 + T2, p = 0.048) had significantly shorter DFS when they co-expressed either positive cleaved caspase-3 or/and higher caspase-3 compared to those who had negative cleaved caspase-3 and lower caspase-3 expression (Fig 6), implying that the roles of cleaved caspase-3 and caspase-3 might be varied in OTSCC patients, depending on tumor stage and microenvironment.

## Discussion

Caspase cascades play crucial roles in apoptosis and are highly associated with cancer development and prognosis. However, the role of expression and activation of various caspases in tumorigenesis remains a double-edged sword. Low expression levels or inactivation of caspases frequently occur in cancer cells and make the cells resistant to microenvironmental stresses and treatments [19, 20]. Conversely, the overexpression of caspases in dying cells may release growth-stimulating signals to allow the non-apoptotic tumor cells to proliferate and survive under stress conditions [16, 17]. To date, little is known about the role of apoptosis-related caspases in OTSCC patients, particularly in a stratification model. In this study, we evaluated the association of the expression of cleaved caspase-3 and caspase-3/8/9 with tumorigenesis and prognosis using a tissue microarray containing 246 OTSCC samples. The findings obtained from the IHC and statistical analyses are as follows. First, the expression levels of cleaved caspase-3 and caspase-3/8/9 in the tumor tissues were significantly higher than those in the adjacent normal tissues. Second, positive expression of cleaved caspase-3 was associated with poor DFS in OTSCC patients, particularly in those with moderate cell differentiation and lymph node invasion. Third, co-expression of either positive cleaved caspase-3 or higher caspase-3 or both were associated with poor DFS in patients with moderately differentiated tumors and small tumor size. However, co-expression of positive cleaved caspase-3 or/and higher caspase-3 was associated with longer DSS in OTSCC patients with advanced disease, such as poor pathological stage, large tumor size and lymph node invasion. Through stratification analysis, these results suggest that the roles of cleaved caspase-3 and caspase-3 in the prognosis of OTSCC might be varied according to the stages of disease. To the best of our knowledge, stratification analysis to determine the relationship between survival curves and expression levels of cleaved caspase-3/caspase-3 in tumor tissues of OTSCC patients has not been previously reported.

Dying cells or surviving tumor cells release several mitogens, such as Wnt and Hedgehog, to promote the growth of neighboring cells by a process called apoptosis-induced proliferation [21]. Indeed, elevated expression of caspases was observed in tumor tissues of several cancer types, including breast carcinomas [13, 22], pancreatic ductal carcinoma [23], non-small cell lung carcinoma [24], and oral carcinoma [16]. In accordance with previous results, our current study showed that the levels of expression of cleaved caspase-3 and caspase-3/8/9 in tumor tissues were higher than in the tumor adjacent normal tissues in OTSCC patients, suggesting that apoptosis-related caspases were elevated to promote tumor growth. Nevertheless, caspases are crucial enzymes in apoptotic mechanism, and it is not surprising that cells induce caspases to eliminate abnormal cells and avoid cancer development during environmental stresses such as DNA damage [8]. In fact, the level of caspase-3 was found to be reduced in tumor tissues compared with corresponding normal tissue in hepatocellular carcinoma [25], pancreatic cancer [19] and prostate carcinoma [26]. These results indicate that the roles of caspases in tumorigenesis might be varied and likely depend on the type of cancer and complex tumor microenvironment.

Activated caspase-3 cleaves cytosolic calcium-independent phospholipase A2 (iPLA_2_) at Asp513 for its activation in dying tumor cells or tumor stroma in response to radiation [17, 27]. The truncated iPLA_2_ (amino acids 514–860) hydrolyzes phospholipids to generate arachidonic acid, which is the precursor for the synthesis of prostaglandin E2 (PGE_2_) with cyclooxygenase-1/-2 (COX1/2) [28]. PGE_2_ is involved in tumor growth, metastasis and cancer stem cell regeneration [28]. Furthermore, blocking PGE_2_ synthesis or inhibiting caspase-3 sensitizes cancer cells to chemotherapy and radiation therapy [17, 29]. Moreover, caspase-7 is another executioner caspase and has overlapping substrate preference with caspase-3 [30]. A high level of expression of caspase-7 is associated with poor DFS in OSCC patients [16]. In our study, we found that co-expression of either positive cleaved caspase-3 or higher caspase-3 or both in tumors correlated with significantly poor DFS compared to negative expression of cleaved caspase-3 and lower caspase-3 in OTSCC patients. This finding is also consistent in OTSCC patients with moderately and poorly differentiated tumors and small tumors (T1 and T2). Our results can be used to speculate that cleaved caspase-3 and caspase-3 expression in tumor cells might activate iPLA_2_ and release PGE_2_ to promote the survival and rapid proliferation of tumor cells, leading to relapse.

A defect in the expression or activation of caspase-3 is one of the hallmarks of cancer cells, which may allow the tumor cells to avoid stress-induced apoptosis and result in poor prognosis. Low caspase-3 activity is associated with a higher risk for local recurrence in colorectal cancer patients [31]. Low expression levels of caspase-3 are associated with poor prognosis in non-muscle-invasive bladder cancer [32]. High levels of cleaved caspase-3 in tumor tissues or stromal cells are associated with good prognosis in colorectal cancer patients [20]. Our stratification results indicate that co-expression of positive cleaved caspase-3 or/and higher caspase-3 are associated with longer DSS in OTSCC patients with advanced disease, including AJCC pathological stage (III and IV), T stage (T3 and T4) and N stage (N1 and N2). Nutrient deprivation and hypoxia are common features in tumors and invasive tumor cells. The activation of caspase-3 is easier and results in apoptosis of cancer cells under both nutrient deficiency and hypoxia conditions [33–35]. These findings might explain why the high co-expression of cleaved caspase-3 and caspase-3 was associated with longer DSS in OTSCC with only large tumors (T3 and T4) and with lymph node invasion (N1 and N2). Nevertheless, the sample size of advanced OTSCC cases with low levels of co-expression of cleaved caspase-3 and caspase-3 is not large enough (n = 4 or 6) in this study; thus our findings need to be validated in a large OTSCC cohort in the near future.

Although caspases might have non-apoptotic functions in tumorigenesis and poor prognosis, our results suggest that cleaved caspase-3, caspase-3, caspase-8 and caspase-9 can serve as biomarkers for tumorigenesis in OTSCC patients. Additionally, co-expression of positive cleaved caspase-3 or/and higher caspase-3 can have oncogenic or tumor suppressive properties in OTSCC patients with tumors of certain stages or differentiation stages. These findings might shed light on the modulation of caspase-3 for the treatment of OTSCC patients in the future.

## Supporting information

S1 Table Clinicopathologic outcomes and survival of patients with OTSCC. (DOC)

S2 Table Distribution of caspases expression in OTSCC. (DOC)

S3 Table Clinical data and caspase expression scores of OTSCC. (XLS)

## Abbreviations

AHR: adjusted hazard ratio
AJCC: American Joint Committee on Cancer
CI: confidence interval
CTAN: corresponding tumor adjacent normal
DFS: disease-free survival
DSS: disease-specific survival
IHC: immunohistochemistry
OTSCC: oral tongue squamous cell carcinoma
TMA: tissue microarray

## References

[1] Kumar S. Regulation of caspase activation in apoptosis: implications in pathogenesis and treatment of disease. Clin Exp Pharmacol Physiol. 1999;26(4):295–303. Epub 1999/05/04. . 10225139 10.1046/j.1440-1681.1999.03031.x

[2] Loro L, Vintermyr OK, Johannessen AC. Apoptosis in normal and diseased oral tissues. Oral Dis. 2005;11(5):274–87. Epub 2005/08/27. doi: 10.1111/j.1601-0825.2005.01117.x . 16120113 10.1111/j.1601-0825.2005.01117.x

[3] Fan CF, Xu HT, Lin XY, Yu JH, Wang EH. A multiple marker analysis of apoptosis-associated protein expression in non-small cell lung cancer in a Chinese population. Folia Histochem Cytobiol. 2011;49(2):231–9. Epub 2011/07/12. . 21744322 10.5603/fhc.2011.0032

[4] Negrini S, Gorgoulis VG, Halazonetis TD. Genomic instability—an evolving hallmark of cancer. Nature reviews Molecular cell biology. 2010;11(3):220–8. doi: 10.1038/nrm2858 . 20177397 10.1038/nrm2858

[5] Mehlen P, Puisieux A. Metastasis: a question of life or death. Nature reviews Cancer. 2006;6(6):449–58. doi: 10.1038/nrc1886 . 16723991 10.1038/nrc1886

[6] Fulda S. Tumor resistance to apoptosis. International journal of cancer. 2009;124(3):511–5. doi: 10.1002/ijc.24064 . 19003982 10.1002/ijc.24064

[7] Fulda S, Pervaiz S. Apoptosis signaling in cancer stem cells. The international journal of biochemistry & cell biology. 2010;42(1):31–8. doi: 10.1016/j.biocel.2009.06.010 . 19577660 10.1016/j.biocel.2009.06.010

[8] Hassan M, Watari H, AbuAlmaaty A, Ohba Y, Sakuragi N. Apoptosis and molecular targeting therapy in cancer. BioMed research international. 2014;2014:150845 doi: 10.1155/2014/150845 . 25013758 10.1155/2014/150845 PMC4075070

[9] McIlwain DR, Berger T, Mak TW. Caspase functions in cell death and disease. Cold Spring Harbor perspectives in biology. 2013;5(4):a008656 doi: 10.1101/cshperspect.a008656 . 23545416 10.1101/cshperspect.a008656 PMC3683896

[10] Reed JC. Mechanisms of apoptosis. Am J Pathol. 2000;157(5):1415–30. Epub 2000/11/14. doi: 10.1016/S0002-9440(10)64779-7 . 11073801 10.1016/S0002-9440(10)64779-7 PMC1885741

[11] Nagata S. Apoptosis by death factor. Cell. 1997;88(3):355–65. Epub 1997/02/07. . 9039262 10.1016/s0092-8674(00)81874-7

[12] Xia B, Yu YH, Guo QS, Li XY, Jiang L, Li J. Association of Fas-670 gene polymorphism with inflammatory bowel disease in Chinese patients. World J Gastroenterol. 2005;11(3):415–7. Epub 2005/01/08. . 15637757 10.3748/wjg.v11.i3.415 PMC4205351

[13] O'Donovan N, Crown J, Stunell H, Hill AD, McDermott E, O'Higgins N, et al Caspase 3 in breast cancer. Clin Cancer Res. 2003;9(2):738–42. Epub 2003/02/11. . 12576443

[14] Fulda S. Caspase-8 in cancer biology and therapy. Cancer Lett. 2009;281(2):128–33. Epub 2008/12/30. doi: 10.1016/j.canlet.2008.11.023 . 19111387 10.1016/j.canlet.2008.11.023

[15] Andressakis D, Lazaris AC, Tsiambas E, Kavantzas N, Rapidis A, Patsouris E. Evaluation of caspase-3 and caspase-8 deregulation in tongue squamous cell carcinoma, based on immunohistochemistry and computerised image analysis. J Laryngol Otol. 2008;122(11):1213–8. Epub 2008/05/27. doi: 10.1017/S0022215108002636 . 18501034 10.1017/S0022215108002636

[16] Coutinho-Camillo CM, Lourenco SV, Nishimoto IN, Kowalski LP, Soares FA. Caspase expression in oral squamous cell carcinoma. Head & neck. 2011;33(8):1191–8. doi: 10.1002/hed.21602 . 21755562 10.1002/hed.21602

[17] Huang Q, Li F, Liu X, Li W, Shi W, Liu FF, et al Caspase 3-mediated stimulation of tumor cell repopulation during cancer radiotherapy. Nature medicine. 2011;17(7):860–6. doi: 10.1038/nm.2385 . 21725296 10.1038/nm.2385 PMC3132290

[18] Liu X, He Y, Li F, Huang Q, Kato TA, Hall RP, et al Caspase-3 promotes genetic instability and carcinogenesis. Molecular cell. 2015;58(2):284–96. doi: 10.1016/j.molcel.2015.03.003 . 25866249 10.1016/j.molcel.2015.03.003 PMC4408780

[19] Jakubowska K, Guzinska-Ustymowicz K, Famulski W, Cepowicz D, Jagodzinska D, Pryczynicz A. Reduced expression of caspase-8 and cleaved caspase-3 in pancreatic ductal adenocarcinoma cells. Oncology letters. 2016;11(3):1879–84. doi: 10.3892/ol.2016.4125 . 26998093 10.3892/ol.2016.4125 PMC4774510

[20] Noble P, Vyas M, Al-Attar A, Durrant S, Scholefield J, Durrant L. High levels of cleaved caspase-3 in colorectal tumour stroma predict good survival. British journal of cancer. 2013;108(10):2097–105. doi: 10.1038/bjc.2013.166 . 23591201 10.1038/bjc.2013.166 PMC3670501

[21] Ryoo HD, Bergmann A. The role of apoptosis-induced proliferation for regeneration and cancer. Cold Spring Harbor perspectives in biology. 2012;4(8):a008797 doi: 10.1101/cshperspect.a008797 . 22855725 10.1101/cshperspect.a008797 PMC3405855

[22] Vakkala M, Paakko P, Soini Y. Expression of caspases 3, 6 and 8 is increased in parallel with apoptosis and histological aggressiveness of the breast lesion. Br J Cancer. 1999;81(4):592–9. Epub 1999/11/26. doi: 10.1038/sj.bjc.6690735 . 10574243 10.1038/sj.bjc.6690735 PMC2362889

[23] Satoh K, Kaneko K, Hirota M, Toyota T, Shimosegawa T. The pattern of CPP32/caspase-3 expression reflects the biological behavior of the human pancreatic duct cell tumors. Pancreas. 2000;21(4):352–7. Epub 2000/11/15. . 11075989 10.1097/00006676-200011000-00005

[24] Tormanen-Napankangas U, Soini Y, Kahlos K, Kinnula V, Paakko P. Expression of caspases-3, -6 and -8 and their relation to apoptosis in non-small cell lung carcinoma. Int J Cancer. 2001;93(2):192–8. Epub 2001/06/19. doi: 10.1002/ijc.1315 . 11410865 10.1002/ijc.1315

[25] Fujikawa K, Shiraki K, Sugimoto K, Ito T, Yamanaka T, Takase K, et al Reduced expression of ICE/caspase1 and CPP32/caspase3 in human hepatocellular carcinoma. Anticancer Res. 2000;20(3B):1927–32. Epub 2000/08/06. . 10928128

[26] Winter RN, Kramer A, Borkowski A, Kyprianou N. Loss of caspase-1 and caspase-3 protein expression in human prostate cancer. Cancer Res. 2001;61(3):1227–32. Epub 2001/02/28. . 11221855

[27] Zhao X, Wang D, Zhao Z, Xiao Y, Sengupta S, Xiao Y, et al Caspase-3-dependent activation of calcium-independent phospholipase A2 enhances cell migration in non-apoptotic ovarian cancer cells. The Journal of biological chemistry. 2006;281(39):29357–68. doi: 10.1074/jbc.M513105200 . 16882668 10.1074/jbc.M513105200

[28] Nakanishi M, Rosenberg DW. Multifaceted roles of PGE2 in inflammation and cancer. Seminars in immunopathology. 2013;35(2):123–37. doi: 10.1007/s00281-012-0342-8 . 22996682 10.1007/s00281-012-0342-8 PMC3568185

[29] Kurtova AV, Xiao J, Mo Q, Pazhanisamy S, Krasnow R, Lerner SP, et al Blocking PGE2-induced tumour repopulation abrogates bladder cancer chemoresistance. Nature. 2015;517(7533):209–13. doi: 10.1038/nature14034 . 25470039 10.1038/nature14034 PMC4465385

[30] Cullen SP, Martin SJ. Caspase activation pathways: some recent progress. Cell death and differentiation. 2009;16(7):935–8. doi: 10.1038/cdd.2009.59 . 19528949 10.1038/cdd.2009.59

[31] de Heer P, de Bruin EC, Klein-Kranenbarg E, Aalbers RI, Marijnen CA, Putter H, et al Caspase-3 activity predicts local recurrence in rectal cancer. Clinical cancer research: an official journal of the American Association for Cancer Research. 2007;13(19):5810–5. doi: 10.1158/1078-0432.CCR-07-0343 . 17908973 10.1158/1078-0432.CCR-07-0343

[32] Wang J, Zhang X, Wei P, Zhang J, Niu Y, Kang N, et al Livin, Survivin and Caspase 3 as early recurrence markers in non-muscle-invasive bladder cancer. World journal of urology. 2014;32(6):1477–84. doi: 10.1007/s00345-014-1246-0 . 24595485 10.1007/s00345-014-1246-0

[33] Li S, Zhang HY, Wang T, Meng X, Zong ZH, Kong DH, et al BAG3 promoted starvation-induced apoptosis of thyroid cancer cells via attenuation of autophagy. The Journal of clinical endocrinology and metabolism. 2014;99(11):E2298–307. doi: 10.1210/jc.2014-1779 . 25062457 10.1210/jc.2014-1779

[34] Mukai M, Kusama T, Hamanaka Y, Koga T, Endo H, Tatsuta M, et al Cross talk between apoptosis and invasion signaling in cancer cells through caspase-3 activation. Cancer research. 2005;65(20):9121–5. doi: 10.1158/0008-5472.CAN-04-4344 . 16230365 10.1158/0008-5472.CAN-04-4344

[35] Nagarajah NS, Vigneswaran N, Zacharias W. Hypoxia-mediated apoptosis in oral carcinoma cells occurs via two independent pathways. Molecular cancer. 2004;3(1):38 doi: 10.1186/1476-4598-3-38 . 15613236 10.1186/1476-4598-3-38 PMC544893

