# Supplementary material for: Expression levels of cleaved caspase-3 and caspase-3 in tumorigenesis and prognosis of oral tongue squamous cell carcinoma
Source: PLoS One. 2017 Jul 10;12(7):e0180620. doi: 10.1371/journal.pone.0180620 (PMC5503265; doi:10.1371/journal.pone.0180620)
Supplement: S1 Table — (DOC) [file pone.0180620.s001.doc]

| **Table S1.** Clinicopathologic outcomes and survival of patients with OTSCC (base of tongue was excluded). | | | |
| --- | --- | --- | --- |
| Variable | No. (%) | CHR (95% CI) | *p value*^＊^ |
| Sex |  |  |  |
| Female | 29 (11.8) | 1.00 |  |
| Male | 217 (88.2) | 1.86 (0.90–3.81) | 0.093 |
| Age, y |  |  |  |
| ≦40 | 47 (19.1) | 1.00 |  |
| 41-50 | 79 (32.1) | 1.63 (0.95–2.81) | 0.077 |
| 51-60 | 67 (27.2) | 1.22 (0.68-2.19) | 0.497 |
| ＞60 | 53 (21.5) | 0.91 (0.48-1.74) | 0.780 |
| Cell differentiation |  |  |  |
| Well | 26 (10.6) | 1.00 |  |
| Moderate | 203 (82.5) | **2.63 (1.15–6.03)** | **0.023** |
| Poor | 17 (6.9) | **7.03 (2.60–18.98)** | **<0.001** |
| AJCC pathological stage |  |  |  |
| I | 72 (29.3) | 1.00 |  |
| II | 95 (38.6) | **2.01 (1.12–3.60)** | **0.019** |
| III | 46 (18.7) | **3.84 (2.07–7.12)** | **<0.001** |
| IV | 33 (13.4) | **7.77 (4.12–14.66)** | **<0.001** |
| T classification |  |  |  |
| T1 | 76 (30.9) | 1.00 |  |
| T2 | 118 (48.0) | **2.04 (1.22–3.39)** | **0.006** |
| T3 | 36 (14.6) | **3.05 (1.65–5.64)** | **<0.001** |
| T4 | 16 (6.5) | **5.29 (2.57–10.88)** | **<0.001** |
| N classification |  |  |  |
| N0 | 195 (79.3) | 1.00 |  |
| N1 | 24 (9.8) | **2.79 (1.64–4.75)** | **<0.001** |
| N2 | 27 (11.0) | **4.42 (2.69–7.27)** | **<0.001** |
| Postoperative RT |  |  |  |
| No | 181 (73.6) | 1.00 |  |
| Yes | 65 (26.4) | **1.89 (1.28–2.79)** | **0.001** |
| Postoperative CT |  |  |  |
| No | 241 (98.0) | 1.00 |  |
| Yes | 5 (2.0) | **4.97 (1.82–13.58)** | **0.002** |
| *Abbreviations: AJCC, American Joint Committee on Cancer; CHR, crude hazard ratio; RT, radiotherapy; CT, c*hemotherapy.  ^＊^*p values were estimated by Cox’s regression.* | | | |
