# Supplementary material for: Expression levels of cleaved caspase-3 and caspase-3 in tumorigenesis and prognosis of oral tongue squamous cell carcinoma
Source: PLoS One. 2017 Jul 10;12(7):e0180620. doi: 10.1371/journal.pone.0180620 (PMC5503265; doi:10.1371/journal.pone.0180620)
Supplement: S2 Table — (DOC) [file pone.0180620.s002.doc]

**S2 Table. Distribution of caspases expression in OTSCC**

| **CC-3** | | | | | |  |
| --- | --- | --- | --- | --- | --- | --- |
| Score | Number | | Percentage | Cumulative percentage | |  |
| 0.00 | 95 | 38.6 | | | 38.6 |  |
| 1.00 | 56 | 22.8 | | | 61.4 |  |
| 2.00 | 57 | 23.2 | | | 84.6 |  |
| 3.00 | 32 | 13.0 | | | 97.6 |  |
| 4.00 | 5 | 2.0 | | | 99.6 |  |
| 5.00 | 1 | .4 | | | 100.0 |  |
| Total | 246 | 100.0 | | |  |  |
| **C-3** | | | | | |  |
| Score | Number | | Percentage | | Cumulative percentage |  |
| 0.00 | 5 | 2.0 | | | 2.0 |  |
| 2.00 | 20 | 8.1 | | | 10.2 |  |
| 3.00 | 21 | 8.5 | | | 18.7 |  |
| 4.00 | 42 | 17.1 | | | 35.8 |  |
| 5.00 | 67 | 27.2 | | | 63.0 |  |
| 6.00 | 86 | 35.0 | | | 98.0 |  |
| 7.00 | 5 | 2.0 | | | 100.0 |  |
| Total | 246 | 100.0 | | |  |  |
| **C-8** | | | | | |  |
| Score | Number | | Percentage | | Cumulative percentage |  |
| 5.00 | 21 | 8.5 | | | 8.5 |  |
| 6.00 | 51 | 20.7 | | | 29.3 |  |
| 7.00 | 174 | 70.7 | | | 100.0 |  |
| Total | 246 | 100.0 | | |  |  |
| **C-9** | | | | | | |
| Score | Number | | Percentage | | Cumulative percentage |  |
| 2.00 | 3 | 1.2 | | | 1.2 |  |
| 3.00 | 11 | 4.5 | | | 5.7 |  |
| 4.00 | 37 | 15.0 | | | 20.7 |  |
| 5.00 | 68 | 27.6 | | | 48.4 |  |
| 6.00 | 78 | 31.7 | | | 80.1 |  |
| 7.00 | 49 | 19.9 | | | 100.0 |  |
| Total | 246 | 100.0 | | |  |  |
